# Insights into grapevine defense response against drought as revealed by biochemical, physiological and RNA-Seq analysis

**DOI:** 10.1038/s41598-017-13464-3

**Published:** 2017-10-13

**Authors:** Muhammad Salman Haider, Cheng Zhang, Mahantesh M. Kurjogi, Tariq Pervaiz, Ting Zheng, Chaobo Zhang, Chen Lide, Lingfie Shangguan, Jinggui Fang

**Affiliations:** College of Horticulture, Nanjing Agricultural University, Tongwei Road 6, Nanjing, 210095 P.R. China

## Abstract

Grapevine is an important and extensively grown fruit crop, which is severely hampered by drought worldwide. So, comprehending the impact of drought on grapevine genetic resources is necessary. In the present study, RNA-sequencing was executed using cDNA libraries constructed from both drought-stress and control plants. Results generated 12,451 differentially expressed genes (DEGs), out of which 8,021 genes were up-regulated, and 4,430 were down-regulated. Further physiological and biochemical investigations were also performed to validate the biological processes associated with the development of grapevine in response to drought stress. Results also revealed that decline in the rate of stomatal conductance, in turn, decrease the photosynthetic activity and CO_2_ assimilation in the grapevine leaves. Reactive oxygen species, including stress enzymes and their related proteins, and secondary metabolites were also activated in the present study. Likewise, various hormones also induced in response to drought stress. Overall, the present study concludes that these DEGs play both positive and negative roles in drought tolerance by regulating various biological pathways of grapevine. Nevertheless, our findings have provided valuable gene information for future studies of abiotic stress in grapevine and various other fruit crops.

## Introduction

Grapevine (*Vitis vinifera* L.) is an important crop, having 7.8 million hectares of cultivated land with an annual production of 67.6 million tons worldwide^1^. The climate change has noticeable effects on the survival and productivity of fruit plants^2^. Hence, the growth of grapevine is consequently, affected by abiotic stress, such as drought and salinity. Among these, drought has deleterious impacts on viticulture around the world^3,4^. Globally, 45% of the agricultural terrains are under constant/periodic water scarcities^5^, causing nearly 50% of yield losses. Plants as sessile organisms can make versatile vicissitudes in physiology and morphology that allow them to endure environmental stress. However, these adaptations are derisory to recover physiological water potential in the cell^6^. Plant response to these minimal water conditions is mediated by expression of numerous genes encoding stress-related proteins, enzymes and metabolites functioning in the various pathways of cell metabolism^7^. The genes induced by osmotic stress in plants are categorized into two groups, such as functional proteins and regulatory proteins^8,9^. In previous studies, several salient genes were identified in grapevine genome in response to biotic and abiotic stresses, 59 similar genes encoding putative WRKY proteins were identified from the available database^10^. Similarly, plant pathogenesis-related proteins were believed to be involved in plant defense and are usually induced during biotic and abiotic stresses^11^. In addition, over-expression of *AtHSP70* has demonstrated its contribution in enhancing tolerance to abiotic stress^12^. Transcriptomic analysis of grapevine during drought stress is of vital importance whose results could provide a defense-related gene information, which offers a foundation for further development of drought-resistant grapevine cultivars.

Water scarcity is not the only threat to viticulture productivity, but also for wine quality^13,14^. Schultz proposed that an increase in environmental temperature due to rise in atmospheric CO_2_ is a primary cause of water shortages for viticulture^15^. Grapevine possesses distinct molecular machinery which adjusts the circulation of water to leaf and then to the atmosphere by vessel anatomy^16^, stomatal conductance^17^ and aquaporin^18^. Consequently, the sluggish leaf and shoot growth, elongation of tendrils, restrained internodes extension, leaf augmentation, a decline of an average diameter of xylem vessels and a minor stimulation in root growth under drought are observed in grapevine^16^.

RNA-seq is a deep-sequencing technology to acquire transcriptomic profiling of both model and non-model plants. In a single assay, this approach reveals the detection of unique genes, transcript information, allele-specific gene expression and single nucleotide variants without the availability of ESTs and gene annotations. Moreover, transcriptome data have also been utilized in defining large-scale genes governing the complex interaction and metabolic processes of the plants under stress^19^. In addition, qRT-PCR enables the quantification of absolute gene expression, which allows researchers to validate the RNA-seq results. Thus, the advantage of this technique ought to be manipulated in the crop production, especially the countries suffered more losses from adverse environmental conditions. we have effectively implicated this technique in our previous study of different fertilization trials in grapevine^20^. However, drought-regulated stress-response in grapevine has not been investigated in detail so far. Therefore, the aim of this study is to elucidate the physiological responses of grapevine to drought stress and further identify and analyze the DEGs in various biological pathways to gain insight into grapevine defense response to drought stress.

## Results

The sequence data obtained from the Illumina deep-sequencing was submitted to Short Read Archive (SRA) database at NCBI under accession number SAMN04914490. After filtering, raw data yielded 42.47 and 53.05 million clean reads in control and drought-stressed leaf samples, respectively. The sequence alignment (soap2/SOAPaligner; http://soap.genomics.org.cn) to the grapevine reference genome, allowed two base mismatch. The total mapped reads (73.44%) were corresponding to unique (72.01%) and multiple (1.44%) genomic positions (Supplementary Table S1).

In the current study, the sum of 12,451 DEGs was expressed under drought stress (|log_2_Ratio| ≥ 1) and false discovery rate (FDR ≤ 0.001); whereas, 8,021 (64.43%) were up-regulated and 4430 (35.57%) were down-regulated (Supplementary Table S2).

### Gene ontology (GO) and KEGG analysis of differentially-expressed genes

A total of 12,451 DEGs were subjected to GO and KEGG for annotation. All the DEGs were assigned to different groups based on their functional properties, such as biological process (21), cellular component (16) and molecular functions (14). Under the broad category of “Biological process”, 4,537 transcripts were assigned to ‘metabolic processes’ (GO: 0008152), followed by ‘cellular process’ which consists of 3,632 transcripts (GO: 0009987) and 3,185 transcripts were involved in ‘single organism process’ (GO: 0044699). Similarly, under “Cellular component” category, 3,247 transcripts were assigned to ‘cell’ and ‘cell part’ (GO: 0005623, and GO: 0044464), followed by 2,358 transcripts in ‘organelle’ (GO: 0043226). Further, under “Molecular functions” category, 4,291 transcripts were in ‘catalytic activities’ (GO: 0003824), and 3,497 transcripts were assigned to ‘binding activities’ (GO: 0005488; Supplementary Table S3; Figure S1 **)**.

Several DEGs from the current study were subjected to KEGG annotation for further characterization of transcripts, where 12,451 transcripts were allotted to 306 KEGG pathways. Our results revealed that the highest numbers of transcripts (1,425) were involved in the “Metabolic pathway” (1,257 up-regulated, 168 down-regulated), followed by “Biosynthesis of secondary metabolites”, which consists of 1,160 transcripts (681 up-regulated, 479 down-regulated), then 756 transcripts were recorded in “Plant-pathogen interaction pathway” (487 up-regulated, 269 down-regulated), while lowest transcripts (21) were recorded in “Sesquiterpenoid and triterpenoid biosynthesis pathway” in which 14 transcripts were up-regulated and 7 transcripts were down-regulated (Supplementary Table S4).

### Chlorophyll degradation and photosynthetic competencies under drought stress

The results of chlorophyll (chl) estimation unveiled that 34.88% decrease of chl-a content in drought treated grapevine leaf (0.28 ± 0.06 mg g^−1^) when compared with that of control plant leaf (0.43 ± 0.11 mg g^−1^). Similarly, 21.92% decrease in chlb of leaf exposed to drought stress (0.57 ± 0.04 mg g^−1^) compared to control leaf (0.73 ± 0.06 mg g^−1^). In the same way, photosynthesis rate was also decreased by 32.20% in drought treatment (16.08 ± 0.75 µmole m^2−^ sec^−1^) when compared to control (23.67 ± 0.81 µmole m^−2^ sec^−1^). Moreover, stomatal conductance and CO_2_ assimilation rate also showed a significant reduction by 40.00% (0.11 ± 0.04) and 44.44% (5 ± 0.03) in drought treated grapevine leaves compared to that of control (Table 1).

**Table 1:** Comparison of physiological and biochemical parameters in grapevine leaves in response to drought stress.

Physiological and biochemical parameters	Control	Drought treatment	Range of increasing %
Chlorophyll contents (mg g^−1^)	1.16 ± 0.08	0.85 ± 0.09	−26.72
Chla contents (mg g^−1^)	0.43 ± 0.11	0.28 ± 0.06	−34.88
Chlb contents (mg g^−1^)	0.73 ± 0.06	0.57 ± 0.04	−21.92
Photosynthesis activity (µmole m^−2^sec^−1^)	23.67 ± 0.81	16.08 ± 0.75	−32.20
Stomatal conductance (µmole m^−2^sec^−1^)	0.15 ± 0.03	0.09 ± 0.02	40.00
Net CO2 assimilation (µmole m^−2^sec^−1^)	9 ± 0.03	5 ± 0.03	44.44
MDA contents (nmol/g)	5.35 ± 0.21	8.61 ± 0.25	60.93
SOD activity (U/g/min)	371.56 ± 10.21	650.85 ± 15.7	75.16
POD activity (U/g/min)	18.23 ± 0.97	43.9 ± 1.01	140.81
CAT activity (U/g/min)	6.32 ± 1.21	19.01 ± 0.99	200.79
Proline (ng/g FW)	1.124 ± 0.04	1.711 ± 0.05	52.37

**Figure 1:**
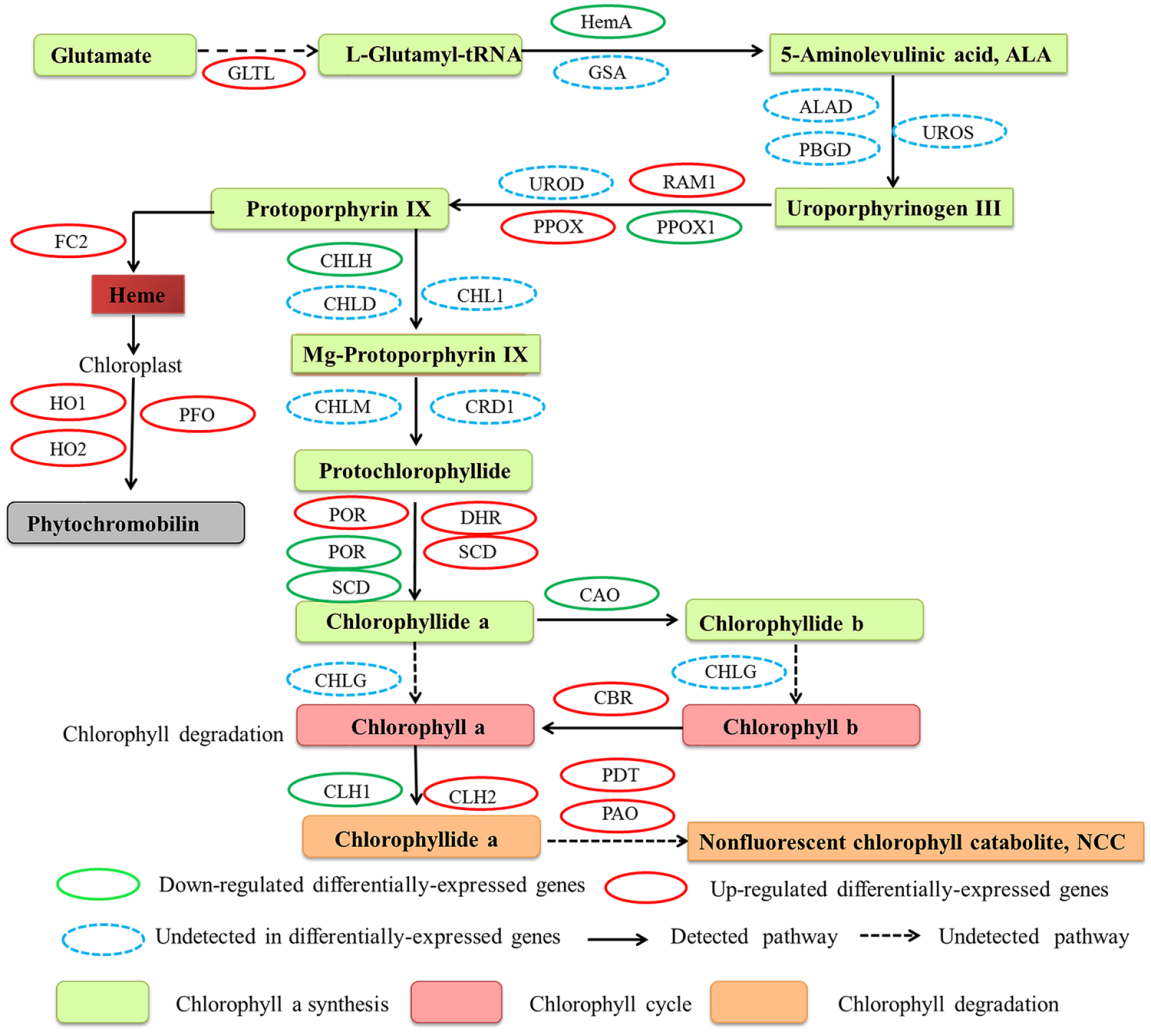
Chlorophyll metabolic pathway in drought-stress grapevine leaves. GLTL, Glutamate tRNA ligase; HemA, Glutamate tRNA reductase 1; GSA, Glutamate-1-semialdehyde; ALAD, Delta-aminolevulinic acid dehydrates; PBGD, porphobilinogen deaminase; UROS, Uroporphyrinogen III synthase; RMA1, Radical S-adenosyl methionine domain-containing protein 1; PPOX1; Proporphynogen oxidase 1; PPOX, Proporphynogen oxidase; UROD, Uroporphyrinogen III decarboxylase; CHLH, Magnesium chelatase H subunit; CHL1, Magnesium-chelatase I subunit; CHLD, Magnesium chelatase D subunit; CHLM, Mg-proto IX methyltransferase; CRD1, Mg-protophyrin IX monomethylester (oxidative) cyclase; POR, Protochlorophyllide oxidoreductase; DHR, Dehydrogenase/reductase SDR family member; SCD, Short chain dehydrogenase, TIC32; CHLG, CAO; Chlorophyllide a oxygenase; CBR, Chlorophyll(ide) b reductase NYC1; CLH1, Chlorophyllase-I; CLH2, Chlorophyllase-II; PAO, Pheophorbide a oxygenase; PDT, Protochlorophyllide-dependent translocon component 52.

In the grapevine transcriptome, 23 DEGs involving in chlorophyll metabolic pathway responded differently to drought stress compared with control, of which 13 transcripts were up-regulated, and 10 transcripts were down-regulated. However, out of 23 transcripts functioning in chl synthesis and degradation, 9 transcripts involved in chla synthesis (Glutamate tRNA Ligase; Radical S-adenosyl methionine domain-containing protein1; Protoporphyrinogen oxidase; Dehydrogenase/reductase SDR family member; Protochlorophyllide oxidoreductase, and four transcripts of Short chain dehydrogenase, TIC32) were significantly up-regulated; whereas, 6 transcripts (2 transcripts of HemA, Glutamate tRNA reductase 1; Proporphynogen oxidase 1; 2 transcripts of CHLH, Magnesium chelatase H subunit; Protochlorophyllide oxidoreductase and Short chain dehydrogenase) were significantly down-regulated. Meanwhile, 1 transcript (Chlorophyllide a oxygenase; CAO) was significantly down-regulated, but 1 transcript (Chlorophyll (ide) b reductase NYC1; CBR) was up-regulated by the drought treatment in the chl cycling process. Whereas, 3 transcripts (Chlorophyllase-II, Pheophorbide a oxygenase, and Protochlorophyllide-dependent translocon component 52) were significantly up-regulated and 3 transcripts of Chlorophyllase-I were down-regulated during the chl degradation process (Table 2, Fig. 1). The expression level of VIT_08s0007g08540.t01 (307.93–106.65 RPKM) and VIT_19s0014g03160.t01 (1360.37–307.58 RPKM) revealed high profusion in chla synthesis pathway. Moreover, in the phytochromobilin synthesis, the expression of Ferrochelatase-2 (VIT_07s0031g03200.t01, |log_2_FC| = 3.032), Heme oxygenase 1 (VIT_11s0016g05300.t01, |log_2_FC| = 2.403), Heme oxygenase 2 (VIT_18s0001g11040.t01, |log_2_FC| = 2.249) and Phytochromobilin:ferredoxin oxidoreductase (VIT_06s0009g03770.t01, |log_2_FC| = 1.705) was also induced by drought stress (Supplementary Table S5).

**Table 2:** List of differentially-expressed genes related to chlorophyll degradation and photosynthesis in response to drought stress.

Trait name	Description	No. of up-regulated	No. of down-regulated	sum
Chlorophyll Metabolism	Chlorophyll a synthesis	9	6	15
	Chlorophyll cycle	1	1	2
	Chlorophyll degradation	3	3	6
Photosystem II	psbB	0	2	2
	psbC	0	2	2
	psbW	0	1	1
Photosystem I	psaB	0	2	2
Cytochrome b6-f complex	petA	0	1	1
	petC	1	2	3
Phtosynthetic electron transport	petF	0	2	2
	petH	2	0	2
F-type ATPase	ATPF1B	0	1	1
	ATPF1A	0	1	1
	ATPF1G	0	1	1
	ATPF0C	0	1	1
Photosynthesis-antenna proteins	LHCB1	0	1	1
	LHCB2	0	1	1
	LHCB3	0	1	1
	LHCB6	0	1	1

psbB, Photosystem II CP47 chlorophyll apoprotein gene; psbC, Photosystem II CP43 chlorophyll apoprotein gene; psbW, Photosystem II reaction center W protein; psaB, photosystem I P700 apoprotein A2 gene; petA, cytochrome f; petC, cytochrome b6-f complex iron-sulfur subunit 1; petF, ferredoxin-3; petH, ferredoxin–NADP reductase, leaf-type isozyme; ATPF1B, ATP synthase CF1 beta; ATPF1A, ATP synthase CF1 alpha; ATPF1G, ATP synthase gamma; LHCB1, chlorophyll a-b binding protein of LHCII; LHCB2, light harvesting chlorophyll A/B binding protein; LHCB3, light-harvesting chlorophyll binding protein 3 gene; LHCB6, chlorophyll a-b binding protein CP24 10 A.

In grapevine transcription analysis, a sum of 23 DEGs related to photosynthesis pathway, including PSII (5), PSI (2), cytochrome b6-f complex (4), photosynthetic electron transport (4), F-type ATPase (4), photosynthesis-antenna proteins (4) were sensitive to drought stress. In PSII (5 DEGs), which includes psbB (2), psbCs (2) and psbW (1) and all 5 DEGs were found to be significantly down-regulated. psbC (VIT_00s0396g00010.t01, 280.56–1.32 RPKM) possessed the high expression abundance. Moreover, 2 psaBs in PSI and 3 transcripts related to cytochrome b6-f complex (petA and petC) revealed a significant reduction in their expression levels, perhaps 1 transcript of petC were found to be increased with the control group. Similarly, 4 genes involved in the photosynthetic electron transport unveiled that, two transcripts of petF (VIT_12s0035g00270.t01 and VIT_06s0080g00410.t01) were down-regulated and two transcripts of petH (VIT_04s0023g03510.t01 and VIT_10s0003g04880.t01) were up-regulated when compared with control. In addition, the F-type ATPase-related genes (ATPF1B, ATPF1A, ATPF1G and ATPF0C) and photosynthesis-antenna proteins-related genes (LHCB1, LHCB2, LHCB3 and LHCB6) were found to be significantly down-regulated in drought treated leaves (Table 2, Supplementary Table S6).

### ROS system under drought stress

**Figure 2:**
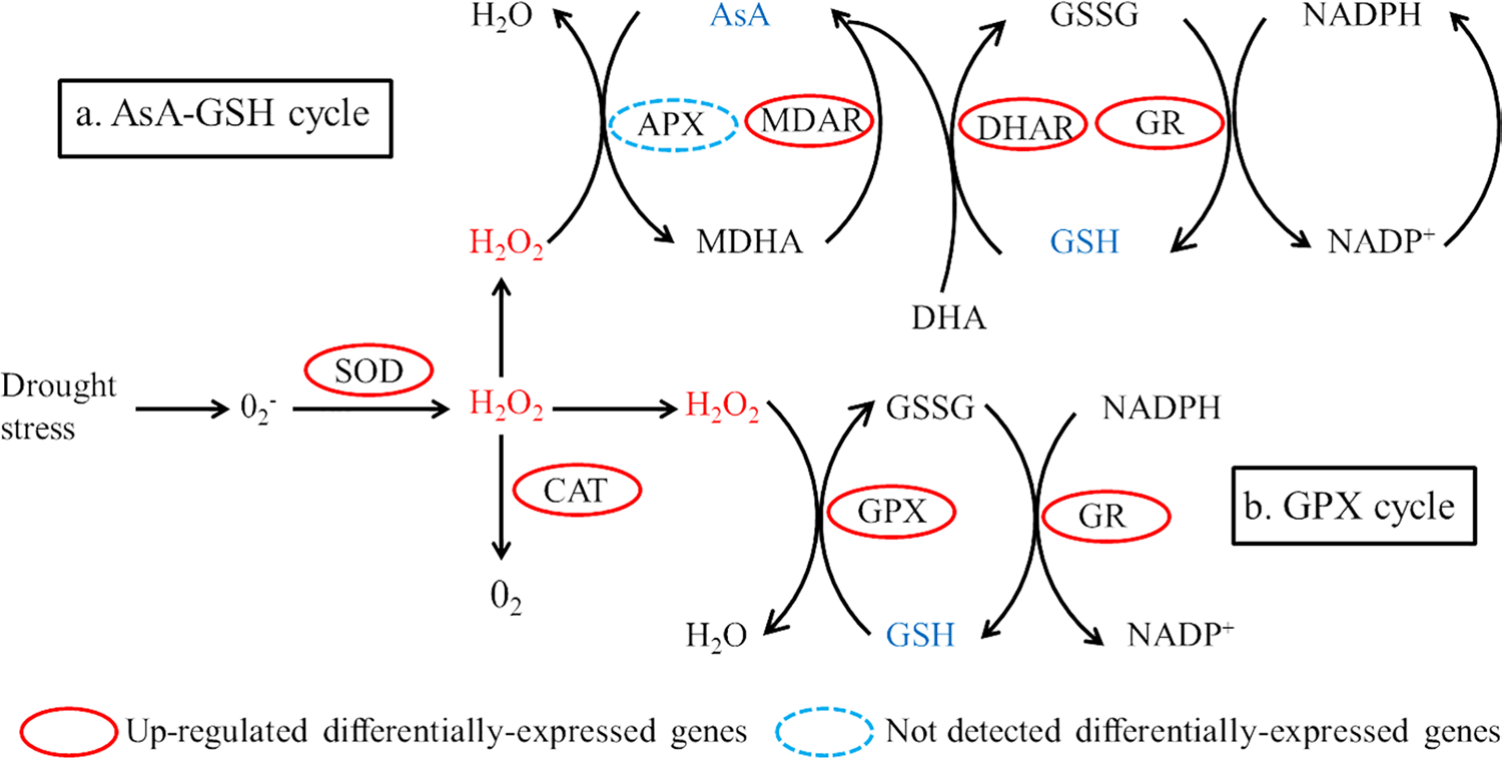
Reactive oxygen species (ROS) scavenging pathway in plants. (**a**) The ascorbate-glutathione (AsA-GSH) cycle, (**b**) The glutathione peroxidase (GPX) cycle. SOD (superoxide dismutase) initiate the line of defense by converting O_2_^−^ into H_2_O_2_, which is further detoxified by CAT (catalses), APX (ascorbate peroxidases (APX) and GPX (glutathione ascorbate). Abbreviations: DHA, dehydroascorbate; GSH, glutathione; GSSG, oxidized glutathione; GR, glutathione reductase; MDAR, monodehydroascorbate reductase; DHAR, dehydroascorbate reductase.

The Malondialdehyde activity was increased significantly (60.93%) in drought treatment (8.61 ± 0.25 nmol g^−1^) compared to control (5.35 ± 0.21 nmol g^−1^). A significant increase in the activity of superoxide dismutase (75.16%), peroxidase (140.81%) and catalase (200.79%) was observed in drought-responsive grapevine in comparison with control (Table 1). In the transcriptomic analysis, one NADPH respiratory oxidase and five amine oxidases functioning in the ROS synthesis process were significantly up-regulated in drought treated grapevine leaf samples. In ROS scavenging system, 60 DEGs were identified and categorized into Fe superoxide dismutase (2 transcripts), peroxidase (6 transcripts), catalase (3 transcripts), glutathione-ascorbate cycle (9 transcripts), glutathione peroxidase (1 transcript), glutathione S-transferase (26 transcripts), peroxiredoxin/thioredoxin pathway (8 transcripts), alternative oxidases (3 transcripts) and polyphenol oxidase (2 transcripts) (Fig. 2; Supplementary Table S7).

In our findings, two Fe-SODS were up-regulated, but both genes showed low expression abundance. In contrast, 2 Cu/Zn-SOD were significantly down-regulated (|log2FC| < 1), CAT (3 transcripts) and POD (2 transcripts) were significantly up-regulated. All three up-regulated CAT transcripts (VIT_18s0122g01320.t01, from 2888.01 to 358.79 RPKM; VIT_00s0698g00010.t01, from 428.21 to 106.90 RPKM; VIT_04s0044g00020.t01, from 767.21 to 263.73 RPKM) showed high expression abundance; whereas, all up/down-regulated POD genes showed moderate to low expression abundance. Furthermore, 9 GSH-AsA (5 up-regulated, 4 down-regulated), 27 GPX-pathway (23 up-regulated, 4 down-regulated), eight Prx/Trx (5 up-regulated, 3 down-regulated), three AOX (2 up-regulated, 1 down-regulated) and two PPO (down-regulated) genes were identified in response to drought stress, (Table 3, Supplementary Table S7).

**Table 3:** List of differentially-expressed genes related to ROS system in response to drought stress.

Trait name	Description	No. of up-regulated	No. of down-regulated	sum
ROS synthesis	Rboh	1	1	2
	AO	5	0	5
ROS scavenging	Fe-SOD	2	0	2
	POD	2	4	6
	CAT	3	0	3
GSH-AsA cycle	MDAR	1	0	1
	DHAR	1	0	1
	GR	1	0	1
	Grx	2	4	6
GPX pathway	GPX	1	0	1
	GST	22	4	26
Prx/Trx	Prx	0	1	1
	Trx	5	2	7
Cyanide-resistant respiration	AOX	2	1	3
Copper-containing enzymes	PPO	0	2	2

Rboh, respiratory burst oxidase; AO, amine oxidase; Fe-SOD, Fe superoxide dismutase; POD, peroxidase; CAT, catalase, APX, ascorbate peroxidase; MDAR, monodehydroascorbate reductase; DHAR, dehydroascorbate reductase; GR, glutathione reductase; Grx, glutaredoxin; GPX, glutathione peroxidase; GST, glutathione S transferase; Prx, peroxiredoxin; Trx, thioredoxin; AOX, alternative oxidase, PPO, polyphenol oxidase.

### Plant hormone signal transduction pathway under drought stress

**Figure 3:**
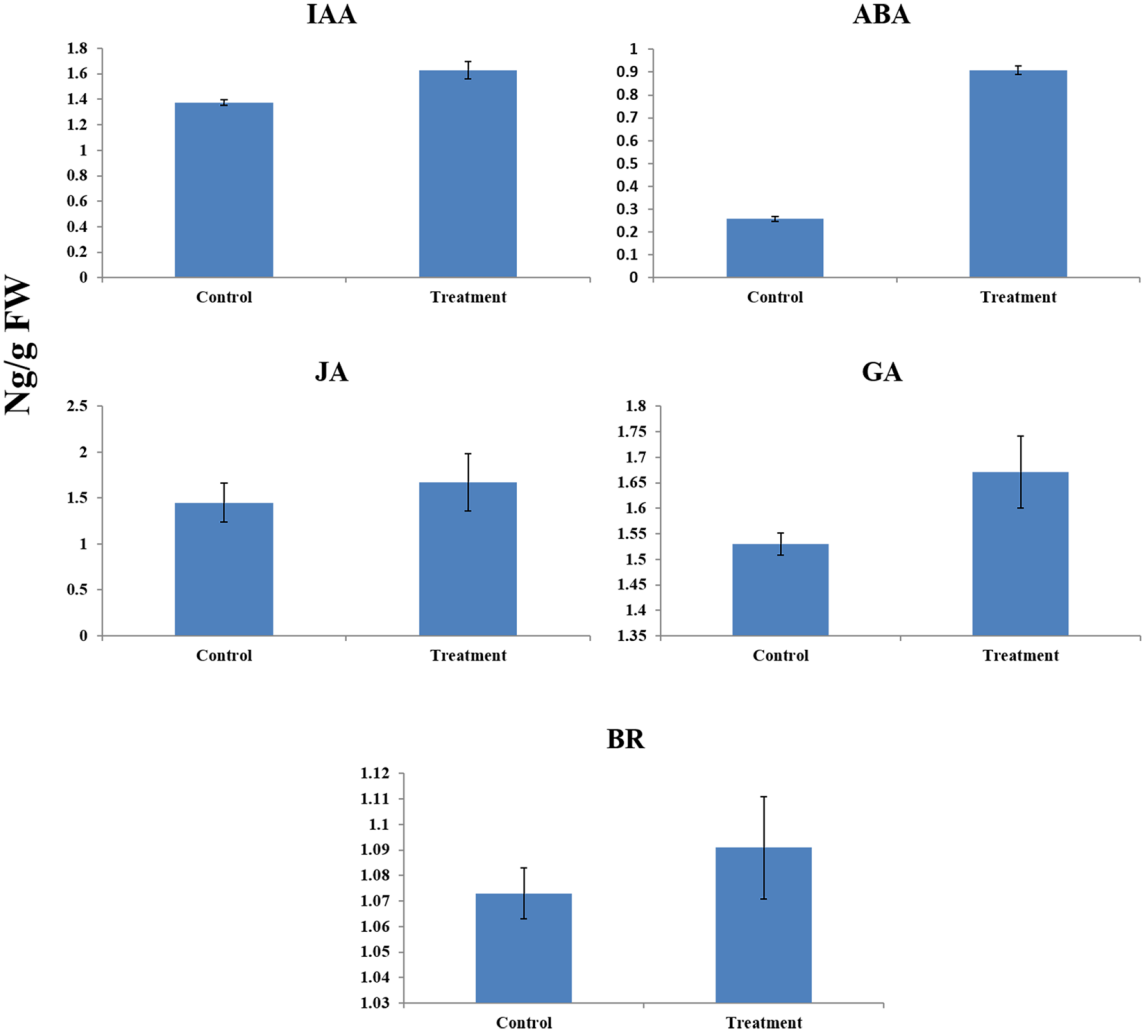
The activities of different hormone, including IAA (indole-acetic acid), ABA (abscisic acid), JA (jasmonic acid), GA (gibberellic acid) and BR (brassinsteroid) in control and drought treatment.

The hormonal level, including auxin, was increased in drought treatment (1.626 ± 0.03 ng g^−1^ FW) compared to control (1.373 ± 0.02 ng g^−1^ FW). A similar trend was observed in abscisic acid that is 0.908 ± 0.01, and 0.257 ± 0.01 ng g^−1^ FW for drought and control treatments, respectively. In the same way, jasmonic acid in drought treatment sample was 1.67 ± 0.05 ng g^−1^ FW, whereas, in control, it was found to be 1.451 ± 0.03 ng g^−1^ FW. Further gibberellic acid (GA) in treated and control sample was recorded to be 1.671 ± 0.02, and 1.53 ± 0.02 ng g^−1^ FW, respectively. Alike brassinosteroid also showed 1.091 ± 0.01, and 1.073 ± 0.01 ng g^−1^ FW for drought and control treatment samples, respectively (Fig. 3). In grapevine transcriptome, several DEGs related to AUX, GA, ABA, JA, ET (ethylene), and BR were found in signal transduction pathways in drought stressed grapevine leaves. Under AUX signaling, three genes (down-regulated) related to auxin transport, eleven auxin-response factors (7 up-regulated and 4 down-regulated) involved in the transcriptional repressors were detected. Moreover, fifteen genes in auxin induced and responsive proteins (2 up-regulated and 13 down-regulated), six IAA synthetase (GH3; 1 up-regulated and 5 down-regulated) and seventeen genes related to auxin and IAA-induced proteins (SAUR; 5 up-regulated and 12 down-regulated) were perceived in grapevine under drought stress. Two natural receptors were up-regulated while four DELLA proteins were down-regulated in the GA under drought stress. Moreover, 3 ABA-responsive proteins (down-regulated), two SNF1-related protein kinases 2 (SnRK2; 1 up- and 1 down-regulated), 3 PP2C group (up-regulated) genes and 6 transcription factors (ABF, up-regulated) were involved in the abscisic acid pathway. Six transcripts of jasmonate-ZIM-domain proteins (one up-regulated and 5 down-regulated) and single jasmonoyl isoleucine conjugate synthase 1 (up-regulated) were found in JA hormonal signaling. Moreover, 12-oxophytodienoate reductase 2-like (up-regulated), linoleate 13S-lipoxygenase 2-1 (up-regulated) and allene oxide synthase (down-regulated) were identified in JA pathway under drought stress. Three ethylene-responsive transcriptional factors (3 up-regulated) being crucial to ET, five ethylene response factor (down-regulated) and three ACC oxidases (up-regulated) were perceived in drought-treated grapevine leaves. In BRs, two transcripts related to BRASSINOSTEROID INSENSITIVE1 (up-regulated), ten (down-regulated) brassinosteroid-regulated proteins (BRU1) and 9 (down-regulated) D-type cyclins were functioning in plant hormone signal transduction pathway under drought stress conditions (Supplementary Table S8).

### Proline metabolism under drought stress

**Figure 4:**
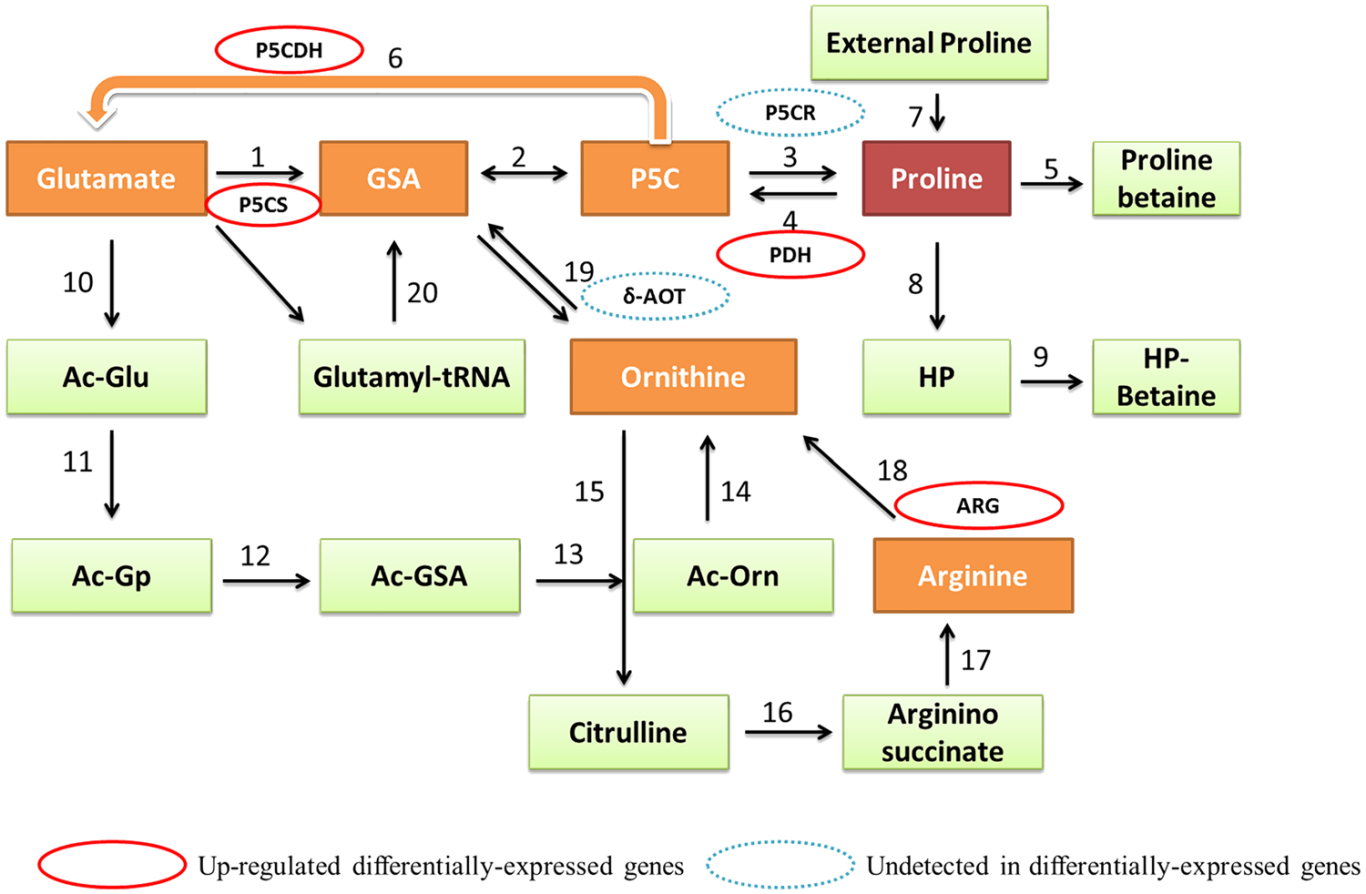
Differential expressions of genes during biosynthesis and degradation of proline in response to drought stress. Given numbers represents the individual genes catalyzing specific reactions. P5CS, pyroline-5-carboxylate synthetase; ARG, arginase; δ-AOT; ornithine-δ-aminotransferase; P5CR, pyrroline 5-carboxylate reductase; PDH, Proline dehydrogenase; P5CDH, Pyrroline-5-carboxyate dehydrogenase.

The proline level showed a significant increase in grapevine leaves responding to drought stress (1.711 ± 0.05 ng g^−1^ FW) as compared with control plant leaves (1.624 ± 0.04 ng g^−1^ FW; Table 1). In transcriptomic analysis, a total of 18 DEGs, including pyroline-5-carboxylate synthetase, proline dehydrogenase, Proline methyltransferase ϒ-Glutamyl kinase, Glutamic-ϒ-semialdehye dehydrogenase, Pyrroline-5-carboxyate dehydrogenase, Prolyl hydroxylase (4 transcripts), Acetyl-CoA: glutamate N-acetyl transferase 2 transcripts), N-Acetylglutamate kinase, Acetyl glutamic-ϒ-semialdehyde dehydrogenase, Acetyl ornithine aminotransferase, Acetyl ornithine deacetylase (2 transcripts), Arginino succinate lyase (ASL) and Arginase were significantly up-regulated functioning in the proline synthesis and metabolism pathway in drought treatment compared to control (Fig. 4, Supplementary Table S9).

### Biosynthesis of secondary metabolites under drought stress

In the transcriptomic study, 70 secondary metabolites related genes linked with shikimate acid (9), alkaloid (2), anthocyanin (32), lignin (19) and terpenoid (8) were recognized under drought treated grapevine leaves.

Shikimate acid (SA) pathway possessed one up-regulated 3-deoxy-D-arabino-heptulosonate-7-phosphate synthase 03, two down-regulated 3-dehydroquinate dehydratase/shikimate dehydrogenase, one down-regulated shikimate kinase one up-regulated chorismate synthase-1, two down-regulated anthranilate phosphoribosyltransferase (AnPRT) and both up-regulated indole-3-glycerol phosphate synthase (IGPS) and tryptophan synthase beta chain 1 (TS1), respectively.

**Figure 5:**
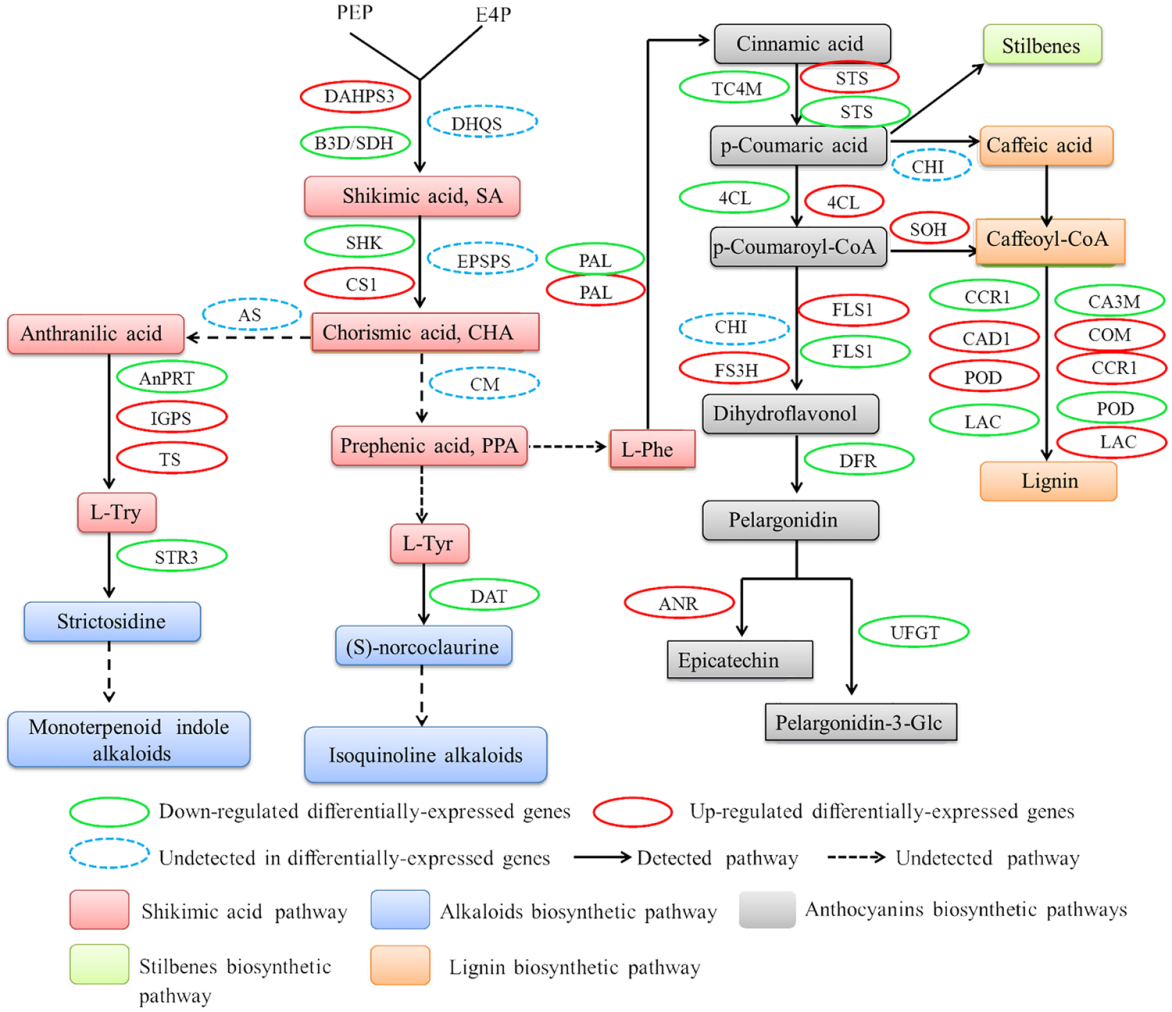
Differential expression of genes related to secondary metabolites under drought stress. DAHPS3, 3-deoxy-D-arabino-heptulosonate-7-phosphate synthase 03; B3D/SDH, bifunctional 3-dehydroquinate dehydratase/shikimate dehydrogenase; SHK, shikimate kinase; CS1, chorismate synthase 1; AnPRT, anthranilate phosphoribosyltransferase; IGPS, indole-3-glycerol phosphate synthase; TS, tryptophan synthase beta chain 1; STR3; strictosidine synthase 3; DAT, D-amino-acid transaminase; PAL, phenylalanine ammonia-lyase; TC4M, Trans-cinnamate 4-monooxygenase; STS, stilbene synthase; 4CL, 4-coumarate–CoA ligase; F3D, flavanone 3-dioxygenase; FLS1, flavonol synthase/flavanone 3-hydroxylase; DFR, dihydroflavonol-4-reductase; UFGT; anthocyanidin 3-O-glucosyltransferase 2; ANR, anthocyanidin reductase; SOH, shikimate O-hydroxycinnamoyltransferase; CA3M, caffeic acid 3-O-methyltransferase; COM, caffeoyl-CoA O-methyltransferase; CCR1, cinnamoyl-CoA reductase 1; CAD1; cinnamyl alcohol dehydrogenase 1; POD; Peroxidase; LAC; laccase.

In alkaloid biosynthetic pathway, genes related to strictosidine synthase 3 and D-amino-acid transaminase were down-regulated. Out of 33 genes in anthocyanin biosynthesis, 8 genes related phenylalanine ammonia-lyase (4-up-regualted and 4-down-regulated), one trans-cinnamate 4-monooxygenase (down-regulated), two 4-coumarate–CoA ligase-like 9 (up and down-regulated), 13 stilbene synthase (6 up-regulated and 7 down-regulated), 3 flavonol synthase/flavanone 3-hydroxylase (one up-regulated and 2 down-regulated), one 1-aminocyclopropane-1-carboxylate oxidase 5 (down-regulated), two dihydroflavonol-4-reductase (down-regulated), one anthocyanidin reductase (up-regulated) and one anthocyanidin 3-O-glucosyltransferase 2 (down-regulated) were observed, (Table 4, Fig. 5, Supplementary Table S10).

**Table 4:** Elucidation on differential expression of genes related to secondary metabolites under drought stress.

Trait name	Description	No. of up-regulated	No. of down-regulated	sum
Shikimate acid pathway	DAHPS3	1	0	1
	B3D/SDH	0	2	2
	SHK	0	1	1
	CS1	1	0	1
	AnPRT	0	2	2
	IGPS	1	0	1
	TS	1	0	1
Alkaloids biosynthetic pathway	STR3	0	1	1
	DAT	0	1	1
Anthocyanin biosynthetic pathway	PAL	4	4	8
	TC4M	0	1	1
	STS	6	7	13
	4CL	1	1	2
	F3D	1	0	1
	FLSI	1	2	3
	DFR	0	2	2
	UFGT	0	1	1
	ANR	1	0	1
Lignin biosynthetic pathway	SOH	1	0	1
	CA3M	0	2	2
	COM	2	0	2
	CCR1	1	1	2
	CAD1	1	0	1
	POD	2	5	7
	LAC	1	3	4
Terpenoid biosynthetic pathway	HMGS	1	1	2
	DXPS	1	1	2
	IPI2	1	0	1
	TSE	1	0	1
	SED	0	2	2

DAHPS3, 3-deoxy-D-arabino-heptulosonate-7-phosphate synthase 03; B3D/SDH, bifunctional 3-dehydroquinate dehydratase/shikimate dehydrogenase; SHK, shikimate kinase; CS1, chorismate synthase 1; AnPRT, anthranilate phosphoribosyltransferase; IGPS, indole-3-glycerol phosphate synthase; TS, tryptophan synthase beta chain 1; STR3; strictosidine synthase 3; DAT, D-amino-acid transaminase; PAL, phenylalanine ammonia-lyase; TC4M, Trans-cinnamate 4-monooxygenase; STS, stilbene synthase; 4CL, 4-coumarate–CoA ligase; F3D, flavanone 3-dioxygenase; FLS1, flavonol synthase/flavanone 3-hydroxylase; DFR, dihydroflavonol-4-reductase; UFGT; anthocyanidin 3-O-glucosyltransferase 2; ANR, anthocyanidin reductase; SOH, shikimate O-hydroxycinnamoyltransferase; CA3M, caffeic acid 3-O-methyltransferase; COM, caffeoyl-CoA O-methyltransferase; CCR1, cinnamoyl-CoA reductase 1; CAD1; cinnamyl alcohol dehydrogenase 1; POD; Peroxidase; LAC; laccase; HMGS, hydroxymethylglutaryl-CoA synthase; DXPS, 1-deoxy-D-xylulose-5-phosphate synthase; IPI2, isopentenyl diphosphate isomerase II; TSE, terpene synthase; SED, squalene epoxidase.

In grapevine transcriptome, 21 differentially expressed genes were identified in lignin biosynthesis, which were involved in drought stress. It includes; 9 up-regulated genes related to shikimate O-hydroxycinnamoyltransferase, aldehyde 5-hydroxylase, two caffeoyl-CoA O-methyltransferase, cinnamoyl-CoA reductase 1, cinnamyl alcohol dehydrogenase 1, two peroxidase and laccase; whereas, 12 DEGs were down-regulated including, two caffeic acid 3 O-methyltransferase, one cinnamoyl-CoA reductase 1, five peroxidases, three laccase transcripts.

Further, eight genes were identified to be involved in terpenoid biosynthesis from which hydroxymethylglutaryl-CoA synthase, 1-deoxy-D-xylulose 5-phosphate reductoisomerase, isopentenyl diphosphate isomerase II and terpene synthase were up-regulated, while hydroxymethylglutaryl-CoA synthase, 1-deoxy-D-xylulose-5-phosphate synthase and two squalene epoxidase were down-regulated in drought-stressed grapevine leaves (Table 4, Fig. 5, Supplementary Table S10).

### Heat shock protein (HSP) and pathogenesis-related protein (PR) in response to drought stress

The results revealed that 49 DEGs were identified in HSPs, including 1 HSP101 (down-regulated), 3 HSP90 (1 up-regulated and 2 down-regulated), 2 HSP70 (1 up-regulated, 1 down-regulated), 18 sHSPs (12 up-regulated and 6 down-regulated), 20 other HSP genes (17 up-regulated and 3 down-regulated) and 5 heat-stress transcription factors (4 up-regulated and 1 down-regulated). The high molecular weight HSPs (HMW HSPs), including HSP90s and HSP70s were also found to be up-regulated in our findings. One up-regulated transcript of HSP70s was expressed at higher abundance level (VIT_17s0000g03310.t01; 650.81 to 532.18 RPKM), compared with other HMW HSPs. The up-regulated, VIT_16s0098g01060.t01 (from 706.59 to 1.98 RPKM) from sHSPs and VIT_14s0060g01490.t01 (from 363.93 to 355.88 RPKM) from other HSPs, expressed at moderate abundances, but remaining other HSPs, along with sHSP, and heat-stress transcription factors expressed at lower abundances (Table 5, Supplementary Table S11).

**Table 5:** List of differentially-expressed genes related to heat-shock proteins (HSPs) and pathogens resistance (PRs) proteins in grapevine perceived during drought stress.

Trait name	Description	No. of up-regulated	No. of down-regulated	sum
Heat shock proteins	HSP101	0	1	1
	HSP90	1	2	3
	HSP70	1	1	2
	small HSP	12	6	18
	other HSP	17	3	20
	heat-stress transcription factor	4	1	5
PR-1	pathogenesis-related protein 2	4	6	10
PR-2	Beta-1,3-glucanase	4	5	9
PR-3, 4, 8, 11	chitinase	4	15	19
PR-5	Thaumatin-like protein	6	8	14
PR-10	Pathogenesis-related protein 10	2	2	4
PR-14	lipid transfer protein	5	5	10
PR-15	germin-like protein 2	2	2	4
PTI	transcriptional activator		2	2
dirigent protein		3	7	10
proline related protein		6	6	12

In this study, 72 DEGs encoding pathogenesis-related proteins were identified, including 10 pathogenesis-related protein PR-1 (4 up-regulated, 6 down-regulated), 9 Beta-1,3-glucanase (PR2; 4 up-regulated, 5 down-regulated), 19 chitinase (4 up-regulated, 15 down-regulated), 14 thaumatin-like protein (PR5; 6 up-regulated, 8 down-regulated), 4 Pathogenesis-related protein-10 (2 up-regulated, 2 down-regulated), 10 non-specific lipid-transfer protein (PR14; 5 up-regulated, 5 down-regulated), 4 Germin-like protein 2 (2 up-regulated, 2 down-regulated), and 2 pathogenesis-related transcription factors (2 down-regulated) to code disease resistance proteins. Conversely to HSPs, most of the PR showed down-regulation in grapevine leaves under drought stress. Moreover, 4 up-regulated transcripts, including PR1 (VIT_03s0088g00890.t01, |log_2_FC| = 8.75), chitinase (VIT_05s0094g00320.t01, |log_2_FC| = 8.29), thaumatin-like protein (VIT_02s0025g04290.t01, |log_2_FC| = 3.84) and Pathogenesis-related protein-10 (VIT_05s0077g01600.t01, |log_2_FC| = 8.31) were only expressed in treatment group. Additionally, 10 dirigent proteins (3 up, 7 down-regulated) and 12 proline related proteins (6 up, 6 down-regulated) were also recorded from this study (Table 5, Supplementary Table S11).

### qRT-PCR validation of DEGs from Illumina RNA-Seq

**Figure 6:**
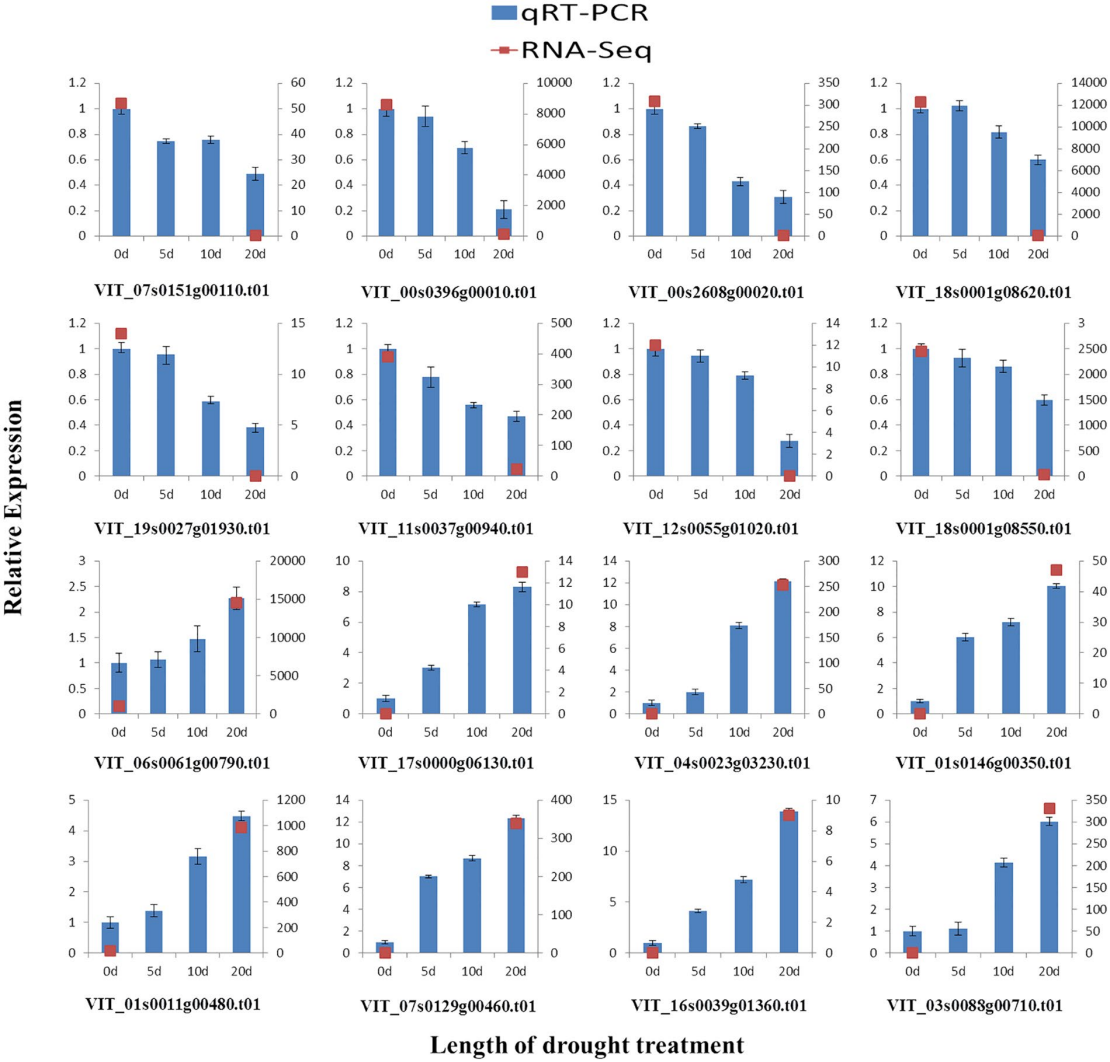
Verification of relative expression levels of DEGs by qRT-PCR. Error bars indicate standard deviation from 3 technical replicates of RT-qPCR. Expression patterns of 16 DEGs selected from different elucidated pathways by qRT-PCR (blue bar) and RNA-Seq (red dot). (1) Seq ID: VIT_07s0151g00110.t01 (Chlorophyllase-1), (2) Seq ID: VIT_00s0396g00010.t01 (psbC; Photosystem II CP43 chlorophyll apoprotein gene), (3) Seq ID: VIT_00s2608g00020.t01 (psbB; Photosystem II CP47 chlorophyll apoprotein gene), (4) Seq ID: VIT_18s0001g08620.t01 (psaB; photosystem I P700 apoprotein A2 gene), (5) Seq ID: VIT_19s0027g01930.t01 (peroxiredoxin (Prx), (6) Seq ID: VIT_11s0037g00940.t01 (S-adenosylmethionine decarboxylase proenzyme), (7) Seq ID: VIT_12s0055g01020.t01 (peroxidase N1-like), (8) Seq ID: VIT_18s0001g08550.t01 (squalene monooxygenase), (9) Seq ID: VIT_06s0061g00790.t01 (Pheophorbide a oxygenase), (10) Seq ID: VIT_17s0000g06130.t01 (glutathione S-transferase U9), (11) Seq ID: VIT_04s0023g03230.t01 (auxin-induced protein 15A-like), (12) Seq ID: VIT_01s0146g00350.t01 (BRASSINOSTEROID INSENSITIVE 1-associated receptor kinase 1), (13) Seq ID: VIT_01s0011g00480.t01 (glutamate 5-kinase), (14) Seq ID: VIT_07s0129g00460.t01 (prolyl 4-hydroxylase 9), (15) Seq ID: VIT_16s0039g01360.t01 (phenylalanine ammonia-lyase), (16) Seq ID: VIT_03s0088g00710.t01 (pathogenesis-related protein PR-1).

In order to investigate the accuracy and reproducibility, 16 DEGs were selected from RNA-Seq results for quantitative real-time PCR; these transcripts represent all the major up/down-regulated functions that were identified in our transcriptome data including, metabolism, hormone signaling, disease resistance and regulatory proteins. The gene function, primer sequence, RPKM, Log_2_ values and qRT-PCR results are presented in Fig. 6; Supplementary Table S12. The qRT-PCR findings of 16 (8 up-regulated and 8 down-regulated) selected genes were consistent with the RNA-seq results, revealing the accuracy and reliability of our RNA-seq results.

## Discussion

Drought stress can subdue the plant growth by hindering many physiological processes of plants. Chlorophylls (chls) are the principal light-absorbing pigments and key components of photosynthesis in plants. The physiological and transcriptomic studies of grapevine leaves responding to drought stress have revealed that chl contents were incredibly decreased, which in turn, inhibited the photosynthetic activity. Similarly, the reduction in chl contents has been reported in corn and chickpea in response to drought stress^21,22^. Moreover, transcriptomic data demonstrated that drought stress suppressed the chl biosynthesis process by inhibiting the activity of key enzymes, such as HemA (Glutamyl-tRNA reductase 1) and CHLH (Magnesium chelatase H subunit), which play an essential role in chla synthesis process^23^. Furthermore, in the chl cycle, the oxygenation reactions of chlorophyll(ide) a to chlorophyll(ide) b were catalyzed by chlorophyllide a oxygenase (CAO)^24^, whose activity likewise was decreased under drought stress, suggesting the inhibition of chl cycle. In contrast, the chlorophyll(ide) b to a conversion is catalyzed by chlorophyll(ide) b reductase NYC1 (CBR) and its activity was up-regulated, suggesting that chl cycle process was also suppressed by the drought treatment^25^. Furthermore, PAO (pheophorbide a oxygenase) is regarded as a vital chl catabolic enzyme^26,27^, and contributed well in chl deprivation process as its activity was enhanced under drought stress (Fig. 1 Supplementary Table S5). Buchert^28^ and Du^29^ have demonstrated the key role of PAO as an important chl degradation enzyme investigated during senescence of broccoli and banana, respectively.

Meantime, the photosynthetic activity, stomatal conductance, and CO_2_ assimilation rate were significantly decreased in drought treated grapevine leaves as compared to control. Similar findings have also been reported in grapevine under Cu and drought stresses^30,31^. Moreover, the photosynthesis-related genes, involved in PSII, PSI, cytochrome b6-f complex, photosynthetic electron transport, F-type ATPase and photosynthesis-antenna proteins were significantly down-regulated by drought stress, yet the extent of light-harvesting proteins (CP47, CP43), which binds the chla molecules was found down-regulated by drought stress (Supplementary Table S6). Furthermore, PsaB is anticipated as the heart of PSI that binds P700 special chlorophyll pair^32^ was also down-regulated by drought stress in our findings. Finally, drought stress gradually decreased the activities of PSII electron transport and light-harvesting complex (photosynthesis-antenna proteins). Stomatal closure is known to reduce the CO_2_ absorption which limits the photosynthetic activity in plants under drought stress environment^33–35^. In the present study, the results of physiology and transcriptome analysis revealed that the decline in photosynthetic process was primarily connected with the chl degradation, which indicates that drought stress has direct influence on the photosynthesis process in plants.

ROS is a universal response of the plants to counter environmental stress to prevent oxidative damage. Several studies have already been performed to investigate the importance of MDA under oxidative stress in different crops, such as wheat (*Triticum aestivum*) and oilseed rape (*Brassica napus*), proposed that MDA was accumulated by drought stress^36–38^. On the contrary, plants have the ability to accrue different antioxidative enzymes to confer drought severity, while similar investigations in olive^39^ and wheat^37^ support our findings of enhanced activity of ROS enzymes against elevated levels of MDA. The transcriptomic investigation revealed that one NADPH oxidase and five amine oxidases were remarkably up-regulated, while both have a crucial role in the ROS synthesis and accumulation under various stress environments^40^. SODs are regarded as the first line of defense against ROS which has two isozymes Fe-SOD and Cu/Zn-SOD in plant chloroplast^40^. It is worth mentioning that Fe-SODs was up-regulated, but Cu/Zn-SODs was down-regulated, which is in agreement with our previous findings in grapevine under Cu stress conditions^30^. Other enzymes, including CAT, POD, GSH-AsA cycle, PPO, GST, AO, MDHAR, DHAR and GR also possess drought-responsive antioxidative defense system in grapevine^41^. Perhaps, non-enzymatic antioxidants, such as glutathione and proline also assisted in enhancing the ROS scavenging system in drought treated grapevine, which is consistent with the ROS scavenging system investigated in the *V*. *vinifera* and *S*. *lycopersicum* under drought stress^42,43^. The observed higher antioxidant capacity, increased activity of ROS enzymes and enhanced expression of genes-related to ROS system seems to be a mechanism operative in plant tolerance to drought stress.

Drought stress causes dehydration in plant cells. Plant hormones, such as abscisic acid, auxin, gibberellin, ethylene, jasmonic acid and brassinosteroid accumulate under dehydration conditions and play a major role in stress tolerance in plants^44^. In *Arabidopsis*, ABA activates the subclass III protein kinases of SnRK2 family, which further facilitate the regulation of stomatal conductance to optimize plant water status through guard cells^45,46^, favor our findings of increased activity of SRK2I protein kinase in drought-treated grapevine. The activation of PP2C genes in grapevine responding to drought stress proposed that PP2C has its primary role in stress tolerance, especially in regulating stomatal responses to cope transpiration losses^47^. The AUX gene family includes early response AUX genes, Aux/IAA, GH3 and SAUR and the regulators of AUX genes, ARF, while their activities were down-regulated in our findings. ARFs are also the important abiotic stress-responsive genes and have their crucial role in physiological processes of fruit plants. Wang *et al*.^44^ investigated the AUX gene family in sorghum (*Sorghum bicolor*) and specified that most of these genes were induced by the exogenous application of IAA under drought stress conditions. Moreover, GA activity and the accumulation of DELLA proteins were up-regulated in drought treatment, while similar findings in *Arabidopsis* have suggested that DELLA proteins restrain the plant growth and promote survival under drought stress^48^. JA biosynthesis and signaling together with ABA and other hormones have been extensively studied in many crops. In current investigations, JA amino acid conjugate (JAR1) was up-regulated, while JAR1 are enduringly present in the plant leaves and together with ABA induce the stomatal closure under osmotic stress, have been extensively studied in *Arabidopsis* ^49^. Interestingly, jasmonate-zim domain proteins (JAZ) were significantly down-regulated, which was observed up-regulated in another study in rice^50^, may be because of prolonged duration of drought stress. Moreover, the activity of AOS and LOX were increased, which is similar to the findings of Leng *et al*.^36^ in *V*. *vinifera*. Ethylene is an important stress hormone because its synthesis is induced under different oxidative environments. Under drought stress, the synthesis of ethylene precursor 1-aminocyclopropane-1-carboxylate oxidase was up-regulated in grapevine, which can stimulate plant development and functioning by prompting the diffusion possibility of ABA to its active site^51,52^. Furthermore, the expressions of the ethylene-related regulatory genes (ETR1 and CTR1) were increased in our findings, suggesting their functional role in ethylene biosynthesis as described by Schachtman and Goodger^53^. BRs are the only plant steroids, which elicit the expression of many genes, especially during stress environments. Brassinosteroid Insensitive 1 (BRI1) was up-regulated in our findings, which is known to play the key role in plant growth, morphogenesis and response to drought stress. Feng, *et al*.^54^ created RNAi mutants for *bdBRI1* in *Brachypodium distachyon* and suggested that this gene produces a dwarf phenotype with enhanced tolerance towards drought stress. BR signal transduction, from cell surface perception to activation of specific nuclear genes will be interesting to investigate in the future.

Plants cope with environmental stress by the accumulation of certain compatible osmolytes, such as proline, which is known to induce drought-tolerance in plants^55^ and up-regulation of all the genes related to proline metabolism is the clear evidence of induced grapevine tolerance in our study. Proline biosynthesis commenced with the phosphorylation of glutamate, which then converted into gulatamic-ϒ-semialdehyde by Pyroline-5-carboxylate synthetase (up-regulated). Similarly, arginine is converted into orthinine by arginase (up-regulated) and then into GSA by the ornithine-δ-aminotransferase (not-detected). GSA is then converted into pyrroline 5-carboxylate (P5C) by spontaneous cyclization. Finally, proline is synthesized from the P5C by P5C reductase (P5CR) enzyme^55,56^. In proline degradation pathway, proline is re-converted into P5C by Proline dehydrogenase (PDH; up-regulated) and then into glutamate by Pyrroline-5-carboxylate dehydrogenase (P5CDH; up-regulated). Thus PDH and P5CDH are believed to be most important enzymes in proline degradation to glutamate^57,58^. Hence, proline metabolism may regulate the gene expression during drought stress.

In higher plants, accumulation of various secondary metabolites, such as amino acids, carbohydrates and lipids occur when a plant is subjected to environmental stress^59^. Shikimate pathway not only acts as a bridge between central and secondary metabolism but also serves as a precursor for other secondary metabolites^60^. Additionally, Tyr is a precursor of IAA and initiate the synthesis of indole alkaloids and isoquinoline alkaloids, which prevent plants from oxidative stress^61^. Phe is considered as the precursor of secondary metabolites family, and PAL participates in phenylpropanoid biosynthesis; a key step towards biosynthesis of stilbenes, flavonoids, lignins and various other compounds^62^. STS (stilbene synthase) catalyzes the initial step of flavonoid biosynthesis pathway, which has the protective function during drought stress^63^. Overall, 4 PAL and 6 STS were significantly up-regulated in our findings, proposing the innate link with drought stress. The respective, up and down-regulation of 1-deoxy-D-xylulose 5-phosphate reductoisomerase and 1-deoxy-D-xylulose-5-phosphate synthase can act as a rate limiting enzymes in MEP pathway, also found in Cu-stressed grapevine leaves^30^. Dimethylallyl diphosphate and isopentenyl diphosphate are the universal 5 carbon precursors found in terpenoid synthesis. It has been reported that one isopentenyl-diphosphate isomerase II can catalyze isopentenyl diphosphate to form dimethylallyl diphosphate and one terpene synthase^64,65^, while both were up-regulated in our findings. The down-regulation of most of the genes related to anthocyanin, lignin and terpenoid biosynthesis have elucidated the negative role of drought stress on an accumulation of secondary metabolites in grapevine leaves.

HSPs are ubiquitous stress-related proteins that act as molecular chaperone, HSP members participate in the protein synthesis, folding, aggregation and transportation from the cytoplasm to different intracellular compartments^66,67^. In the present study, majority of HSP genes were up-regulated (35), however genes related to sHSP along with heat stress transcription factors and other HSP were found to be predominant in their expression, in contrast, it was noted that drought has also significantly suppressed few genes (14) related to HSP. Heat-shock protein 70 (Hsp70) proteins are one of the large families of highly conserved molecular chaperones and are extensively found in almost all organisms. HSP70 is one of the highly conserved protein, known to induce during environmental stress, for example, in *Erianthus arundinaceus*, HSP70 was expressed 7-fold higher under drought-stressed condition^68^. sHSPs are plant tolerant proteins generally induced upon abiotic stress^69^. In the same way, Vasquez reported that expression of HSP70 and sHSP was higher during stress conditions in pine tree, indicating their major role in mitigating the drought stress^70^. Therefore, transcriptional analysis of the present study revealed that HSP also play a fundamental role in defense mechanism of grapevine during drought stress. In addition, pathogenesis-related (PR) proteins are derived from plant allergens and act as defense-responsive proteins by increasing their expression under pathogen attack and variable stress environments. Depending on the functions and properties, PR-proteins are classified into 17 families, such as beta-1,3-glucanases, chitinases, thaumatin-like proteins, peroxidases, small proteins (defensins and thionins) and lipid transfer proteins (LTPs)^11,71^. Most of the PR-proteins were down-regulated in our study, suggesting that drought stress posed a negative effect on PR-proteins defense response. Contrarily, most of the genes related to dirigent-proteins (DIR), play a role in lignin formation and proline-related proteins were up-regulated, suggesting their possible defensive role in grapevine in response to drought stress.

## Conclusion

Our results have provided substantial shreds of evidence to demonstrate that grapevine adaptation to drought stress is a multi-step component system consisting of several genes that regulate various pathways. Out of 12,451 DEGs, 8021 transcripts were up-regulated and 4,430 transcripts were down-regulated. Nearly 2 fold up-regulations of DEGs have clearly indicated their defense-related role in grapevine responding to drought stress. The significant increase in the activity of ROS enzymes and hormones level revealed the defensive function of these enzymes and hormones during drought stress in grapevine leaves. Transcription analysis unveils that drought has affected the overall physiology of the grapevine; however, the regulatory mechanism of certain key genes like chlorophyll synthesis, ROS system, HSPs and other defense-related pathways assist grapevine in mitigating drought severity.

## Materials and Methods

### Plant material and drought treatments

Two-year old grapevine (*V*. *vinifera* cv. ‘Summer Black’) pot grown plants were selected as experimental material which were grown in standard greenhouse condition (25 ± 5 °C) under 16-h light/8-h dark photoperiod and 65% relative humidity (RH) at the Nanjing Agricultural University-Nanjing, China. Grapevine plants were subjected to drought with an interval of 20 days against control, each with three biological replicates. The fourth unfolded leaf from the shoot apex was collected from each replicates of both control and drought treatment with the interval of 0 and 20th day, respectively, and the three samples were mixed to make one composite sample. After harvesting, the samples were immediately put in liquid nitrogen and then stored at −80 °C until analysis.

### Determination of important biochemistry and physiology-related traits

The chlorophyll a and b contents was determined using spectrophotometer at 663 and 645 nm, respectively as briefly explained by Leng, *et al*.^26^. Photosynthesis activity, stomatal conductance and CO_2_ assimilation rate were carried out on mature leaf between 4^th^ to 7^th^ nodes from the shoot base for both control and drought treatment; between 9:00–11:00 AM measured using LI-COR (LI-6400XT, Germany) meter as described by Tombesi *et al*. (2015). Malondialdehyde (MDA) contents were quantified by using thiobartiburic acid. The activities of antioxidant enzymes (SOD, POD and CAT) were measured using the method briefly described by Haider, *et al*.^72^. The activities of indole-acetic acid (IAA), abscisic acid (ABA), jasmonic acid (JA), gibberellic acid (GA) and brassinosteroid (BR) were measured following the method of Tombesi, *et al*.^31^. Three technical repeats were generated for all the quantifications. Data was subjected to one-way analysis of variance (ANOVA) at p < 0.05, using MINITAB (ver. 16) and represented as mean ± standard deviation (SD).

### RNA extraction, cDNA library construction and Illumina deep sequencing

Total RNA from leaf samples of both control and drought-stressed were extracted using Trizol reagent (Invitrogen, Carlsbad, CA, USA) (1% agarose gel buffered by Tris–acetate-EDTA was run to indicate the integrity of the RNA.) and subsequently used for mRNA purification and library construction with the Ultra™ RNA Library Prep Kit for Illumina (NEB, USA) following the manufacturer’s instructions. The samples were sequenced on an Illumina HiseqTM2500 for 48 h.

### Analysis of gene expression level, gene ontology (GO) and Kyoto encyclopedia of genes and genomics (KEGG)

After adaptor trimming and quality trimming, the clean reads were mapped to the *V*. *vinifera* transcriptome using Bowtie 1.1.2. Then, Sam tools and BamIndexStats.jar were used to calculate the gene expression level, and reads per kilobase per million (RPKM) value was computed from SAM files^73^. Gene expression differences between log_2_ and early stationary phase were obtained by MARS (MA-plot-based method with Random Sampling model), a package from DEGseq. 3.3 (Leng *et al*., 2015). We simply defined genes with at least 2-fold change between two samples and FDR (false discovery rate) less than 0.001 as differential expressed genes. Transcripts with |log2FC| < 1 were assumed to have no change in their expression levels. The gene ontology (GO) enrichment (p-value < 0.05) was investigated by subjecting all DEGs to GO database (http://www.geneontology.org/) in order to further classify genes or their products into terms (molecular function, biological process and cellular component) helpful in understanding genes biological functions. Kyoto encyclopedia of genes and genomics (KEGG; the major public pathway-related database) was used to perform pathway enrichment analysis of DEGs^74^.

### Illumina RNA-seq results validation by qRT-PCR

In order to validate the Illumina RNA-seq results, drought-stressed grapevine leaf samples of each collection were applied to qRT-PCR analysis. Total RNA of the collected samples was extracted following the above mentioned method, and then was reverse-transcribed using the PrimeScript RT Reagent Kit with gDNA Eraser (Takara, Dalian, China), following the manufacturers’ protocol. Gene specific qRT-PCR primers were designed using Primer3 software (http://primer3.ut.ee/), for 20 selected genes with the sequence data in 3′UTR (Table S12). qRT-PCR was carried out using an ABI PRISM 7500 real-time PCR system (Applied Biosystems, USA). Each reaction contains 10 µl 2 × SYBR Green Master Mix Reagent (Applied Biosystems, USA), 2.0 µl cDNA sample, and 400 nM of gene-specific primer in a final volume of 20 µl. PCR conditions were 2 min at 95 °C, followed by 40 cycles of heating at 95 °C for 10 s and annealing at 60 °C for 40 s. A template-free control for each primer pair was set for each cycle. All PCR reactions were normalized using the Ct value corresponding to the Grapevine UBI gene. Three biological replicates were generated and three measurements were performed on each replicate.

## Electronic supplementary material

Supplementary: Figure S1

Supplementary: Table S1

Supplementary: Table S2

Supplementary: Table S3

Supplementary: Table S4

Supplementary: Table S5

Supplementary: Table S6

Supplementary: Table S7

Supplementary: Table S8

Supplementary: Table S9

Supplementary Table S10

Supplementary Table S11

Supplementary Table S12
